# A Novel Feature Selection Strategy for Enhanced Biomedical Event Extraction Using the Turku System

**DOI:** 10.1155/2014/205239

**Published:** 2014-04-06

**Authors:** Jingbo Xia, Alex Chengyu Fang, Xing Zhang

**Affiliations:** ^1^College of Science, Huazhong Agricultural University, Wuhan, Hubei 430070, China; ^2^Department of Chinese, Translation and Linguistics, City University of Hong Kong, Kowloon, Hong Kong; ^3^The Halliday Centre for Intelligent Applications of Language Studies, City University of Hong Kong, Kowloon, Hong Kong

## Abstract

Feature selection is of paramount importance for text-mining classifiers with high-dimensional features. The Turku Event Extraction System (TEES) is the best performing tool in the GENIA BioNLP 2009/2011 shared tasks, which relies heavily on high-dimensional features. This paper describes research which, based on an implementation of an accumulated effect evaluation (AEE) algorithm applying the greedy search strategy, analyses the contribution of every single feature class in TEES with a view to identify important features and modify the feature set accordingly. With an updated feature set, a new system is acquired with enhanced performance which achieves an increased *F*-score of 53.27% up from 51.21% for Task 1 under strict evaluation criteria and 57.24% according to the approximate span and recursive criterion.

## 1. Introduction

Knowledge discovery based on text mining technology has long been a challenging issue for both linguists and knowledge engineering scientists. The application of text mining technologies based on large collections of known texts, such as the MEDLINE data base, has become especially popular in the area of biological and medical information processing [1, 2]. However, the rate of data accumulation is ever increasing at an astonishing speed [3]. The published literature grows exponentially and huge amounts of scientific information such as protein property and gene function are widely hidden in prohibitively large collections of text. For example, the literature in PubMed grows at a speed of two printed pages per second. As a result, it is practically impossible to manually curate experimental results from texts. As a result of this information explosion, text mining and linguistic methods have been used to perform automatic named entity recognition (NER) including biomedical NER [4], the extraction of clinical narratives [5] and clinical trials [6], analysis of the similarity in gene ontology [7], and the prioritization of vital function genes [8].

While entity recognition has been exploited as a powerful approach towards automatic NER retrieval, there has recently been an increased interest to find more complex structural information and more abundant knowledge in documents [9]. Hence, as a more recent development, moving beyond the purpose of entity recognition, the GENIA task in the BioNLP 2009/2011 shared tasks [10, 11] was set to identify and extract nine biomedical events from GENIA-based corpus texts [12], including gene expression, transcription, protein catabolism, localization, binding, phosphorylation, regulation, positive regulation, and negative regulation. The GENIA task is a publicly accepted task within the BioNLP communities and it is a leading pioneer in the area of structured event extraction.

Designed by Bjorne et al. [13], the Turku Event Extraction System (TEES) was the leading participant tool in both the BioNLP 2009 and 2011 shared tasks. Among the twenty-four participants of BioNLP shared tasks, TEES ranked first place in the GENIA task in 2009. While Riedel et al. performed best (with an *F*-score of 56.0%) for the GENIA task in 2011 ST [14], TEES was the third for the GENIA task and ranked number one for the other four of the eight subtasks. In 2011 and 2012, Björne et al. [15, 16] enhanced TEES and the *F*-score was increased further from 52.86% to 53.15%. The system was kept updated, and its 1.01 version achieved 55.65% in *F*-score. Meanwhile, TEES was also utilized for practical database searching. The system was applied to all PubMed citations and a number of analyses of the extracted information were illustrated [17–19]. TEES is now available at http://bionlp.utu.fi/. In 2011, the BioNLP shared task expanded from the GENIA task to eight generalized tasks including GENIA task, epigenetics and posttranslational modification task, infectious disease task, bacteria biotope task, and bacteria interaction task [11]. As the only system that participated in all the tasks in 2011, TEES was again ranked first place for four out of the eight tasks. Afterwards, Bjorne and Salakoski updated TEES 1.01 to TEES 2.0 in August 2012. The updated system is now capable of handling the DDI'11 (drug-drug interaction extraction) challenge (http://jbjorne.github.io/TEES/) in addition to all the BioNLP 2011 shared tasks. At the moment, the most up-to-date version is TEES 2.1 released in 2013 [20] and a major change is the wider coverage for serving more XML format corpus, and the core algorithm remains unchanged.

As a sophisticated text mining classifier, TEES uses enormous feature classes. For the BioNLP 2009 shared tasks, it produced 405,165 features for trigger detection and 477,840 features for edge detection out of the training data, which not only consume large amounts of processing time but also create undesirable noises to affect system performance. To address this issue and achieve even better system performance in terms of processing time and recognition accuracy, a natural starting point is to perform an in-depth examination of the system processes for feature selection (FS) and feature reduction (FR).

The integration of FS and FR has been demonstrated to hold good potentials to enhance an existing system [21–24]. Feature reduction can delete redundant features, avoid overfitting, provide more efficient modelling, and help gain a deeper insight into the importance of features. It aims to obtain a subset through an optimization between the feature size and the performance [25]. Amongst all of the feature reduction algorithms, the greedy search algorithm, also called hill climbing algorithm, is such an optimal technique for the identification and selection of important features [26]. van Landeghem et al. [27] introduced a feature selection strategy in the Turku system for a different testing data set and illustrated how feature selection could be applied to better understand the extraction framework. In their scheme, candidate events were trained to create a feature selector for each event type. The classifier was then built with the filtered training samples. By doing this, different trigger words for different event types were discriminated and those individual features with a higher occurrence frequency were assigned higher weights. Tag clouds of lexical vertex walks showed certain properties that are interesting from a linguistic point of view.

The purpose of this paper is to focus on the feature selection rules of TEES and aim to improve its performance for the GENIA task. By designing an objective function in the greedy search algorithm, we propose an accumulated effect evaluation (AEE) algorithm, which is simple and effective and can be used to numerically evaluate the contribution of each feature separately concerning its performance in the combined test. Moreover, we make further changes to the core feature class by incorporating new features and merging redundant features in the system. The updated system was evaluated and found to produce a better performance in both Tasks 1 and 2. In Task 1, our system achieves a higher *F*-score of 53.27% according to the “strict evaluation” criterion and 57.24% according to the “approximate span and recursive” criterion. In Task 2, the new strategy achieved an *F*-score of 51.77% under the “strict evaluation” criterion and 55.79% under the “approximate span and recursive" criterion. These represent the best performance scores till now for event recognition and extraction.

## 2. Materials and Method

### 2.1. GENIA Task and Scheme of Turku TEES System

#### 2.1.1. GENIA Task and the Evaluation Criteria

Different from the previous NER task, the GENIA task aims to recognize both the entities and the event relationship between such entities. Extended from the idea of semantic networks, the recognition task includes the classification of entities (nodes) and their associated events (edges). The participants of the shared task were expected to identify nine events concerning given proteins, that is, gene expression, transcription, protein catabolism, localization, binding, phosphorylation, regulation, positive regulation, and negative regulation. The mandatory core task, Task 1, involves event trigger detection, event typing, and primary argument recognition [10]. An additional optional task, Task 2, involves the recognition of entities and the assignment of these entities. Finally, Task 3 targets the recognition of negation and speculation. Because of their fundamental importance and vast potential for future developments, researchers mainly focus on Tasks 1 and 2.

Like the other text mining systems, the performance of TEES is evaluated by precision, recall, and *F*-score. The first measure equals the fraction of obtained relevant documents and the retrieved documents represent the correctness of the extraction system; that is,

(1) Precision =|{relevant  documents}∩{retrieved  documents}||{retrieved  documents}|.

The second measure, recall, is defined as

(2) $$\begin{array}{c} \text{Recall}=\frac{\left|\left\{\text{relevant}\;\text{documents}\right\}\cap \left\{\text{retrieved}\;\text{documents}\right\}\right|}{\left|\left\{\text{relevant}\;\text{documents}\right\}\right|}. \end{array}$$

Recall is used to assess the fraction of the documents relevant to the query that are successfully retrieved. Precision and recall indicators are well-known performance measures in text mining, while *F*-score is a third measure that combines precision and recall and is the harmonic mean of precision and recall:

(3) $$\begin{array}{c} F\text{-score}=2\cdot \frac{\text{precision}\cdot \text{recall}}{\text{precision}+\text{recall}}. \end{array}$$

The *F*-score evenly weighs the precision and recall and forms a reliable measure to evaluate the performance of a text mining system. For more information, refer to [28].

#### 2.1.2. Scheme of TEES

**Figure 1:**
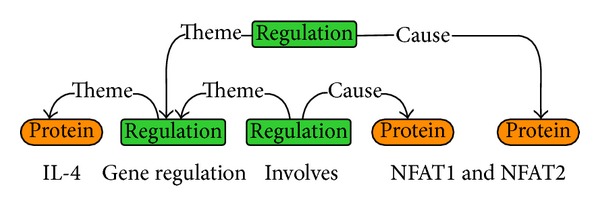
The graphical representation of a complex biological event (refer to [5]).

The core of TEES consists of two components, that is, classification-style trigger/event detection and rich features in graph structure. By mapping the tokenized word and entity to the node and mapping event relation to edge between entities, TEES regards the event extraction as a task of recognizing graph nodes and edges as shown in Figure 1.

Generally, TEES first convert the node recognition to a problem of 10-class classification, which corresponds to 9 events defined in the shared task and another class for negative case. This procedure is defined as trigger detection. Thereafter, the edge detection is defined by the recognition of concrete relationships between entities, including semantic direction and theme/cause relation.

As in Figure 1, the word “IL-4” is assigned to class “protein,” while “involves” is assigned to class “regulation.” The edge between “IL-4” and “regulation” is labelled as “theme.” Hence we obtain a simple regulation event, namely, “IL-4 regulation,” which means “IL-4” is the theme of “regulation.” Similarly, the representation in Figure 1 indicates that the simple event “IL-4 regulation” is the theme of “involves” and the protein “NFAT1” is the cause of “involves,” from which a complex regulation event can be extracted; that is, “IL-4 regulation” regulates protein “NFAT1.” For more detailed information, refer to Bjorne's work [5] and the website of TEES, http://bionlp.utu.fi/eventextractionsoftware.html.

The data set used in TEES consists of four files in GENIA corpus [3]. Each file is given in a stand-off XML format with split sentences, annotation of known proteins, parts of speech (POS), and syntactic dependency tree information. One is train123.xml, which contains 800 abstracts with 7,482 sentences; another is devel123.xml, which contains 150 abstracts with 1,450 sentences. The file everything123.xml is the sum of the two files above, and test.xml is the test data set, which contains 950 abstracts with 8,932 sentences.

**Figure 2:**
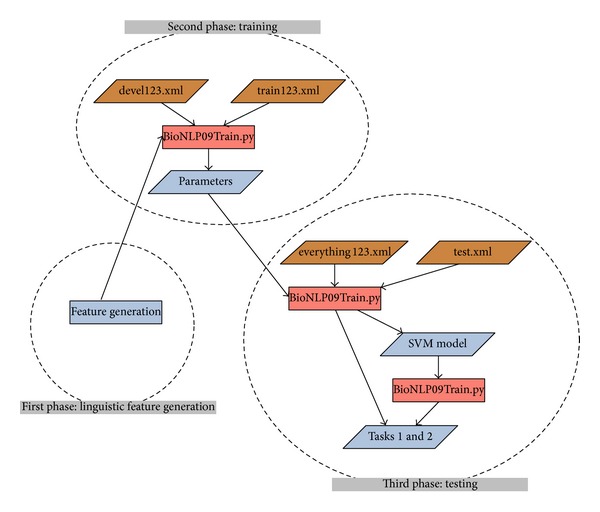
Data set and pipeline of TEES, cited from TEES Toolkit. Location: TurkuEvent ExtractionSystem readme.pdf, p. 7 in the zip file, http://bionlp.utu.fi/static/event-extractor/TurkuEventExtractionSystem-1.0.zip.

The scheme of TEES system consists of three phases. First, linguistic features are generated in the feature generation phase. Second, in the training phase, train123.xml is used as the training data set, devel123.xml is used as development set, and optimum of parameters is obtained. Then in the third phase, using everything123.xml (sum of train123.xml and devel123.xml) as the training set, test.xml is used as unknown data set for event prediction. Events are extracted from this unknown set and accuracy is subsequently evaluated. See Figure 2.

Mainly, there are two parameters in grid searching at the training stage. The first parameter is *C* in polynomial kernel function of support vector machine. Second, in order to set a proper precision-recall trade-off, a parameter *β* (*β* > 0) is introduced in trigger detection. For “no trigger” class, the given classification score is multiplied by *β* so as to increase the possibility of tokens falling into “trigger” class. Optionally, *β* is set as 0.6, and it will be put into grid searching for obtaining optimum value.

### 2.2. Definition of Feature Generation Rule of Turku System

Features used for trigger detection are designed in a rational way, and abundant features are generated from training data set.

In terms of the training data set with GENIA format, the dependency relation of each sentence is output by Stanford parser [29], which addresses the syntactic relations between word tokens and thus converts the sentence to a graph, where the node denotes a word token and the edge corresponds to the grammatical relation between two tokens. By doing this, a directed acyclic graph (DAG) is constructed based on the dependency parse tree, and the shortest dependency path (SDP) is located.

For a targeted word in sentence which represents a node in graph, the purpose of trigger detection is to recognize the event type belonging to the word. Meanwhile, edge detection is to recognize the theme/cause type between two entities. Therefore, both detections can be considered as multiclass classification.

**Figure 3:**
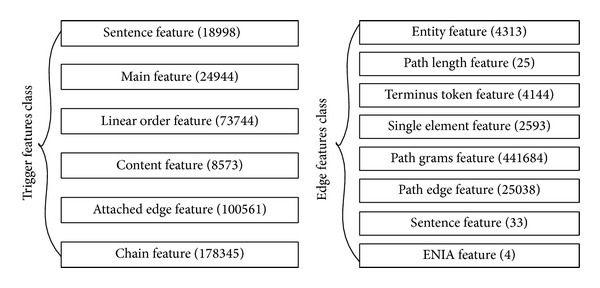
Feature class in trigger detection and edge detection.

The features produced in trigger detection are categorized into six classes, as listed in Figure 3, and the whole feature generation rule is listed in Supplementary Appendix  A in the Supplementary Material available online at http://dx.doi.org/10.1155/2014/205239. First, for a sentence in which the target word occurs, token information is counted for the whole sentence as a bag of words (BOW). This feature class is defined as “sentence feature.” The second feature class is “main feature” of the target word, including part of speech (POS) and stem information output by Porter Stemmer [29]. The third class, “linear order feature,” focuses on the information about the neighboring word tokens in natural order of the sentence. TEES also maintains a fourth class about the microlexical information of the word token, for example, upper or lower case of the word, existence of digital number, double letter in the word, and three letters in the word. These features constitute a “content feature” class, while the “attached edge feature” class focuses on the information about the neighboring word tokens of the target word in SDP. Finally, there is a sixth “chain feature” class, which focuses on the information of the whole SDP instead.

Here, the features are well structured from the macro- and microperspectives about the sentence. Basically, the feature generation rule of “main feature” and “content feature” mostly relies on microlevel information about the word token itself, while “sentence feature” and “linear order feature” rely on the macrolevel information about the sentence. Differently, “attached edge” and “chain feature” rely on the dependency tree and graph information, especially the SDP.

Similarly, features used for edge detection can be classified into 8 classes, namely, entity feature, path length feature, terminus token feature, single element feature, path grams feature, path edge feature, sentence feature, and GENIA feature. Among the features above, the third feature is omitted, since it is considered part of the first feature.

### 2.3. Feature Evaluation Method: Accumulated Effect Evaluation (AEE) Algorithm

A quantitative method is designed to evaluate the importance of feature classes. Here, the feature combination methods are ranked by *F*-score, and the occurrence of *i*th feature class in the top *j*th combinations (*j* runs from the top one to the last one) is counted, the rate of occurrence is computed, and finally the sum of the occurrence is calculated. The total calculation is shown in Supplementary Material Appendix  C. The algorithm is denoted as AEE1 algorithm as shown in Algorithm 1.

Here, AEE1(*i*) is the contribution value of the *i*th feature and also the objective function of the greedy search algorithm, which reflects the accumulated effect of the *i*th feature among the top classifiers. Since AEE1(*i*) makes sense in a comparative way, the theoretical maximum and minimum of AEE1(*i*) are calculated by

(4) $$\begin{array}{c} \text{Max\_AEE}1=1\times 2^{n-1}+\frac{2^{n-1}}{2^{n-1}+1}+\frac{2^{n-1}}{2^{n-1}+2}\cdots +\frac{2^{n-1}}{2^{n}-1},\\ \text{Min\_AEE}1=0\times \left(2^{n-1}-1\right)+\frac{1}{2^{n-1}}+\frac{2}{2^{n-1}+1}\cdots +\frac{2^{n-1}}{2^{n}-1}. \end{array}$$

The idea of AEE1 comes from the understanding that the top classifiers with higher *F*-scores include more efficient and important features. However, this consideration disregards the feature size in terms of those top classifiers.

For better understanding, a simple case is considered. Assume there are two feature classes that could be used in the classifier and so there are three feature combinations for the classifier, namely, 1, 2, and 1&2. Without loss of generality, we assume that the best classifier uses the feature class 1&2, the second one uses the feature class 1, and the worst classifier uses the feature class 2. Here, we denote the rank list 1&2, 1, and 2 as Rank Result A. According to the third column in Table 1(a), AEE value will be calculated as AEE1(1) = 1/1 + 2/2 + 2/3 = 2.667.

As another example, we assume a rank list 1, 1&2, and 2, which is denoted as Rank Result B and shown in Table 1(b). As can be seen from Table 1(b), AEE1(1) equals 2.667, the same as in Table 1(a). However, this is not a reasonable result, since feature 1 is more significant in Rank Result A than in Rank Result B if the feature class size is considered.

Therefore, an alternative algorithm, AEE2, is proposed by updating *c*_*ij*_ = *O*_*ij*_/*j* in Step 4 of AEE1 as *c*_*ij*_ = *O*_*ij*_/∑_*i*=1_^*n*^ *O*_*ij*_. Here, the size of feature class is considered for the computation of *c*_*ij*_ and a classifier with higher performance and smaller feature size will ensure a high score for the feature it owns. As an example, column 4 in Tables 1(a) and 1(b) shows that AEE2(1) = 1.667 in Rank Result A and AEE2(1) = 2.167 in Rank Result B. The result ensures the comparative advantage for feature class 1 in Rank Result A. Similarly, AEE2(*i*) mainly makes sense in a comparative way with the theoretical maximum and minimum values, which are also similarly computed.

Considering AEE1(1) = 2.667 > AEE1(2) = 2.167 and AEE2(1) = 1.667 > AEE2(2) = 1.333 in Table 1(a), the importance of feature class 1 prevails over that of feature class 2 in both cases. This ensures a reasonable consistency. Actually, both AEE1 and AEE2 algorithms ensure a correct weighting rank among different feature classes and a hybrid of the two methods is used in the experiments.

### 2.4. Flowchart of the Research

Unlike a trial-and-error procedure, the scheme of this research is oriented towards better feature selection so that important feature classes are identified by evaluating the contribution of the individual features.

**Figure 4:**
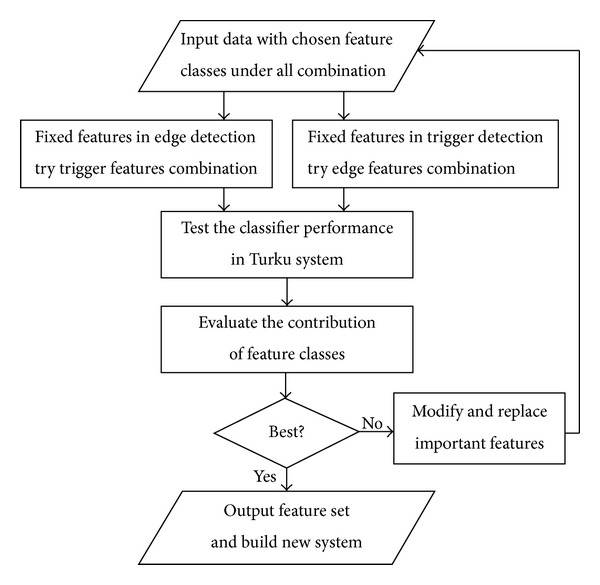
Flowchart of the research.

Accordingly, codes are written to enhance vital features. Thereafter, the classifiers with new updated features are tested and better performance is presumed. Compared with the previous TEES system, our main contribution is to import feature selection strategy in Phase 1, that is, “linguistic feature selection,” as shown in Figure 2. The flowchart of our research is shown in Figure 4.

## 3. Results

### 3.1. Evaluation of Individual Feature Classes by Quantitative Method

Based on the AEE algorithm, all of the combinations of the feature classes are used in classifiers and their corresponding *F*-scores are ranked so as to evaluate the contribution of the individual feature classes.

Using the quantitative algorithm, the importance of feature classes is addressed in the two-phase TEES procedure that involves trigger detection and edge detection. For better understanding, the top ten classifiers with respective feature combinations are shown in Table 2, and the total calculation is shown in Supplementary Material Appendix  B. The final results are collected in Tables 3 and 4.

**Figure 5:**
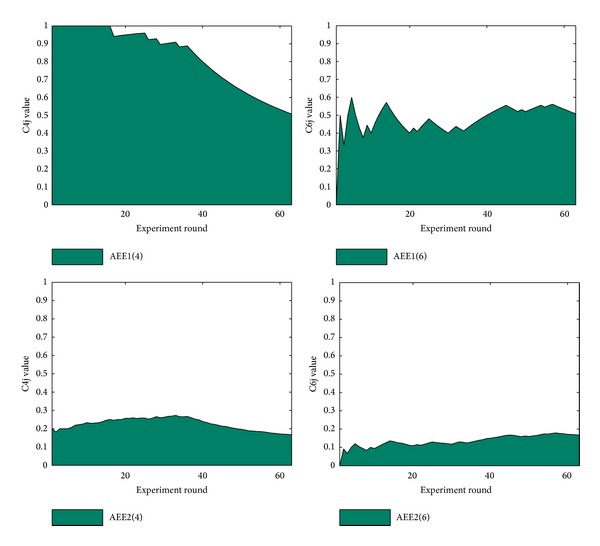
AEE1 and AEE2 plots of the best/worst feature in trigger detection.

**Figure 6:**
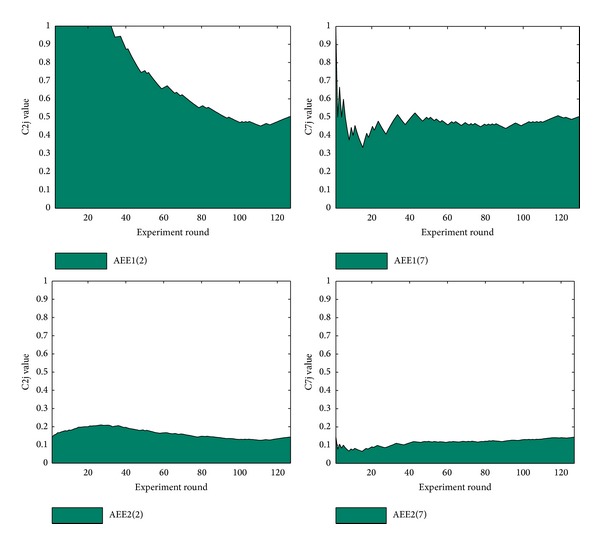
AEE1 and AEE2 plots of the best/worst feature in edge detection.

During the trigger detection, the results show that the 4th feature performs best and 6th feature performs worst. By calculating AEEi value of features, Figures 5 and 6 show plots of the best and the worst feature classes in trigger detection.

The comparison between the two features shows how the 4th feature performs better than 6th feature. And 52.23 and 30.09 also correspond to the value of area below the curve. The AEE1 and AEE2 plot of the best and worst features in trigger and edge detections are shown in Figures 5 and 6.

### 3.2. Modification of Features and New Experiment Results

Combinations of features for trigger detection show that the “content feature” in trigger detection is the most important one, and the “chain feature” is the worst. This result shows that, in terms of identifying a target word token, the target itself provides more information than the neighboring tokens.

Taking the feature generation rule of 4th feature into consideration, the “content feature” class contains four features, “upper,” “has,” “dt,” and “tt.” Specifically, “upper” is to identify the upper case or lower case of letters, “has” is to address the existence of a digit or hyphen, “dt” is to record the continuous double letters, and “tt” is to record three continuous letters. Since the content feature is vital for trigger detection, the feature generation rule could be strengthened similarly. Accordingly, a new “ft” feature is inserted into the “content feature” class by which the consecutive four letters in the word are considered. Moreover, modification is performed on the 6th feature class by merging similar features related to dependency trees in both the 5th and the 6th feature classes. For simplicity, the updated features are denoted as 4′ and 6′.

Furthermore, if we compare the best performance between classifiers with trigger feature comprising the original features, 4′ features, 6′ features, or 4′&6′ features (all include original edge features), we get that the best ones for trigger features are 1&2&3&4&5, 1&2&3&4′&5, 1&2&3&4&5&6′, and 1&2&3&4′&5&6′, respectively, while the *F*-score reaches 51.34, 52.21, 51.93, and 51.99. The new combination result is listed in Supplementary Material Appendix  D.

The complete combination experiment shows that the best combination of trigger feature is 1&2&3&4′&5, with an *F*-score of 52.21. Generally, the best feature combination covers the major part of feature sets. An interesting phenomenon in the best combination is the absence of the 6th or 6′th feature class, which indicates that this feature class is redundant and can be done without it.

Similarly, for feature selection in edge detection, various experiments are carried out based on the best combination of trigger features. Here, the best feature and the worst feature (2nd and 7th feature) are chosen to be modified in edge detection, and we denote the new feature classes as 2′ and 7′. With the fixed trigger feature 1&2&3&4′&5, we test the classifier with the original edge features, 2′ feature, 7′ feature, and 2′&7′ feature. We obtain the best classifier in each combination experiment. The best classifier in each combination owns a feature set 1&2&4&5&6&7&8, 1&2′&4&5&6&7&8, 1&2&4&5&6&7′&8, or 1&2′&4&5&6&7′&8, separately, and the achieved *F*-score is 52.16, 52.37, 52.47, or 52.68, respectively.

In the above experiments, we test the performance of trigger feature class by fixing edge features. Likewise, we test edge features by fixing trigger features. We observe that feature modifications in this phase are indeed capable of achieving improvement, where all of the best combinations perform better than the result of the best trigger. Finally, we use the best trigger feature (1&2&3&4′&5) and best edge feature (1&2′&4&5&6&7′&8), and eventually the best combination of feature set achieved the highest score of 53.27, which is better than the best performance of 51.21 previously reported for TEES 2.0. Therefore, it is concluded that the best classifier has a feature set with trigger feature 1&2&3&4′&5 and edge feature 1&2′&4&5&6&7′&8, where trigger-*c* = 250000, edge-*c* = 28000, and *β* = 0.7. The final result is listed in Table 5.

**Figure 7:**
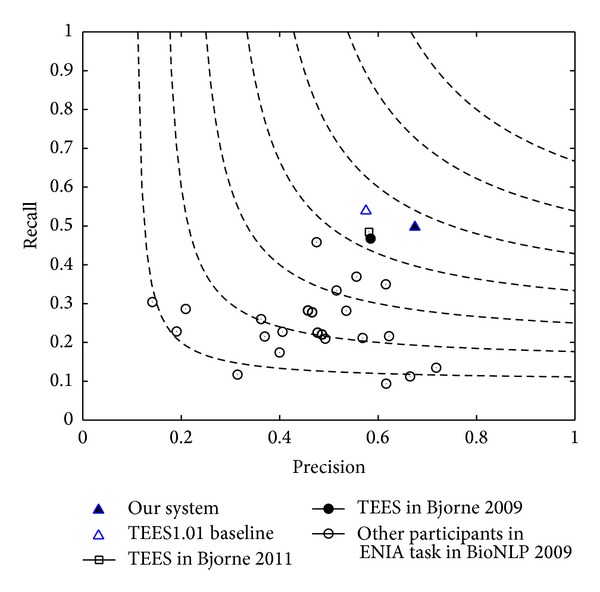
*F*-score contour performance of participants in GENIA task. This *F*-score is evaluated under approximate span and recursive mode. Our current system is marked with a full triangular label.

Comparing with the 24 participants in GENIA task of BioNLP 2009 and historical progress of Bjorne's work, a contour performance is given in Figure 7.

As Figure 6 indicates, our system ranks the first among the 24 systems. Comparisons of *F*-scores are listed in Table 6.

## 4. Discussions and Conclusions

### 4.1. An Analysis of the Contribution of Features

In this research, we designed a feature selection strategy, AEE, to evaluate the performance of individual feature classes to identify the best performing feature sets. An important finding is that the greatest contribution comes from the content feature class in trigger detection. In this section, a routine analysis of the contribution is shown, which yields the same finding and supports the same conclusion that the content feature class contributes the most towards event recognition and extraction.

First, retaining one feature class in the classifier, we can get separate *F*-scores based on these features. Dividing the *F*-score by feature size, we calculate the average contribution roughly. This value partly reflects the contribution by feature classes in terms of class size. The result in Table 6 shows that the average contribution of the 4th feature is 0.003, which is the greatest score achieved by individual feature class.

Second, we observe all of the double combinations involving the *i*th feature and observe that, in most cases, when the *i*th feature is combined with the 4th feature, it reaches the best performance score. Even the worst performance of the double feature combinations involving the 4th feature performs much better than the other settings.

Third, a similar phenomenon occurs in the case of three-feature-combination experiment and four-feature-combination experiment. In all cases, when *i*th feature is combined with the 4th feature, it reaches the best performance. The same as before, even the worst performance of double feature combination involving the 4th feature is much better than the other combinations. See Table 7.

Finally, in yet another analysis, we observe the cases where the *i*th feature is cancelled out. The results show that the combination without the 4th class performs worst, which in turn confirms the importance of the 4th feature.

Through the routine analysis above, there is ample evidence arguing in support of the importance of the 4th feature in trigger detection. Compared with the results in numerical scores, the contribution value of the 4th feature is greater than the others, which confirms the judgment. Furthermore, we can sort these features according to their contribution values. These results can further prove our decision to modify the 4th feature and thereafter enhance system performance.

It is interesting that the routine analysis shows the substantial positive evaluation for the 4th trigger feature, which is proved by the results of quantitative analysis of AEE algorithm. This shows a consistent tendency of feature importance, which in turn proves the reliability of the AEE algorithm. Since it is clumsy to use routine analysis to analyze all of the features, we expect that the AEEi algorithm makes sense in generalized circumstances.

### 4.2. Linguistic Analysis of Chosen Trigger Features and Importance of Microlexical Feature

The effectiveness of the 4th trigger feature motivated the feature class modification by inserting “ft” features. One should also note that all these features in the 4th feature class are related to the spelling of word tokens, which is similar to stems but contains more abundant information than stems. Besides, we can also analyze and ascertain the importance of other features, like POS information, through feature combinations. Here, the edge features are fixed, and only a smaller section of the trigger features is tested through combination experiments.

The full combinations are listed in Supplementary Material Appendix  E and Table 8 lists the top 5 important features. Here, “stem” and “nonstem” are the stem part or left part of stem after using Porter Stemmer [28]. It is also generated in microlexical level, similar to “dt” and “tt.” The results in this table show that the lexical feature generation rule affects the token information extraction in a decisive way.

### 4.3. Strategy Discussion in Feature Selection under Machine Learning Strategy

As a matter of fact, trigger features could be analyzed according to their generation rule, namely, sentence feature, main feature, linear order feature, content feature, attached edge feature, and chain feature. This is a state-of-the-art strategy in feature selection. TEES is a nice system based on machine learning, which, however, does not perform intensive feature selection. The absence of a feature selection strategy in previous research mainly stems from two reasons. The first is that the natural core idea of machine learning is just to put enough features into the classifier as a black box; the second is that the performance of a classifier with huge sizes of features is always better in accuracy and *F*-score. Previously, the features used in TEES have been mostly chosen by trial and error. By adding some codes to produce additional features and seeing how they impact the performance of the system, TEES always achieves a higher *F*-score but with unsure directions. This strategy introduces useful but uncertain features and produces a large number of features. By introducing a feature selection and evaluation method, the importance of different feature classes could be ranked in a proper way, which helps to identify the important features to be modified effectively. Therefore, in terms of the current research regarding the development of the Turku Event Extraction System, we believe that our research reported in this paper is helpful for further improving this system. We also believe that our strategy will also serve as an example for feature selection in order to achieve enhanced performance for machine learning systems in general.

## Supplementary Material

There are five appendix files in the Supplementary Material. Appendix A is the feature generation rule for trigger and edge features. Appendix B is the result of feature combination experiment, which aim to analyze the trigger feature or edge feature contribution. Appendix C is the quantitative algorithm for evaluating contribution of feature class, while Appendix D is the feature combination experiment with modified features. Finally, Appendix E is feature combination experiment with modified features, which targeting on the ontribution analysis of sole trigger or edge feature.

## Figures and Tables

**Algorithm 1:**
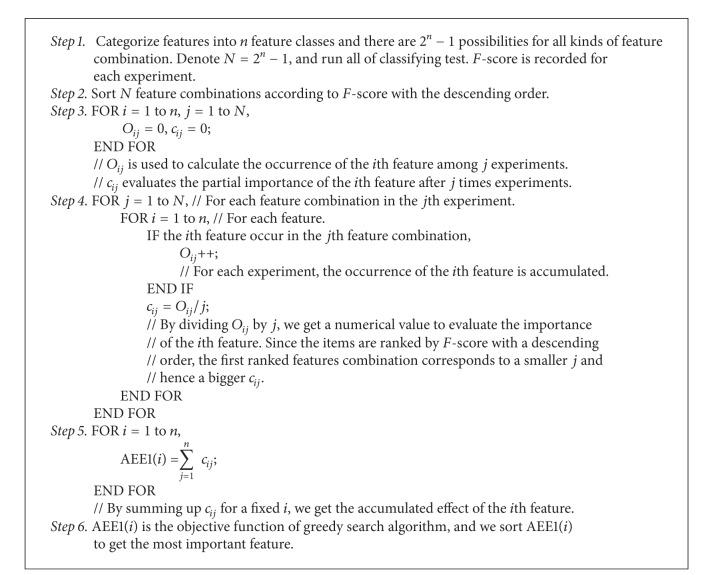
AEE1 algorithm.

**Table:** (a) AEEi result for Ranking Result A

Rank	Result A	*O*_*ij*_			AEE1 *c*_*ij*_			AEE2 *c*_*ij*_	
		*i* = 1	*i* = 2		*i* = 1	*i* = 2		*i* = 1	*i* = 2
*j* = 1	1&2	1	1		1/1	1/1		1/2	1/2

*j* = 2	1	2	1		2/2	1/2		2/3	1/3

*j* = 3	2	2	2		2/3	2/3		2/4	2/4

				AEE1(*i*)	2.667	2.167	AEE2(*i*)	1.667	1.333

**Table:** (b) AEEi result for Ranking Result B

Rank	Result B	*O*_*ij*_			AEE1 *c*_*ij*_			AEE2 *c*_*ij*_	
		*i* = 1	*i* = 2		*i* = 1	*i* = 2		*i* = 1	*i* = 2
*j* = 1	1	1	0		1/1	0/1		1/1	0/1

*j* = 2	1&2	2	1		2/2	1/2		2/3	1/3

*j* = 3	2	2	2		2/3	2/3		2/4	2/4

				AEE1(*i*)	2.667	1.167	AEE2(*i*)	2.167	0.833

**Table 2:** Top 10 best classifiers with corresponding feature classes in combination experiment.

Rank	*F*-score (%)	Trigger feature combination with fixed trigger feature	*F*-score (%)	Edge feature combination with fixed edge feature
1st	51.34	1&2&3&4&5	52.16	1&2&4&5&6&7&8
2nd	51.21	1&2&3&4&5&6	51.81	1&2&4&5&6&8
3rd	50.71	1&2&4&5	51.29	1&2&5&6&7&8
4th	50.19	1&2&3&4&6	51.18	1&2&5&6&8
5th	49.9	1&2&4&5&6	50.33	1&2&4&6&7&8
6th	49.74	1&3&4&5	50.23	1&2&4&6&8
7th	49.44	1&4&5	49.26	2&4&5&6&8
8th	49.16	1&2&3&4	49.02	1&2&4&5&6
9th	48.82	1&2&4&6	48.32	2&4&5&6&7&8
10th	47.82	1&2&4	47.42	2&5&6&8

**Table 3:** Score of features in trigger detection.

Feature ID	1	2	3	4	5	6		

Feature name	Sentence feature	Main feature	Linear order feature	Content feature	Attached edge feature	Chain feature	Theoretical maximum	Theoretical minimum

AEE1 score	40.83	39.94	32.99	52.23	36.12	30.09	53.43	10.27

AEE2 score	10.80	10.82	8.99	14.16	9.80	8.43	20.52	3.28

**Table 4:** Score of features in edge detection.

Feature ID	1	2	4	5	6	7	8		

Feature	Entity	Path length	Single element	Path grams	Path edge	Sentence	GENIA	Theoretical	Theoretical
Name	Feature	Feature	Feature	Feature	Feature	Feature	Feature	Maximum	Minimum

AEE1 score	77.60	83.84	68.45	74.88	77.76	56.09	66.35	107.61	20.08

AEE2 score	18.63	19.57	16.57	17.51	17.88	13.40	15.45	35.80	5.54

**Table 5:** The best feature combination after choosing the best trigger feature (1&2&3&4′&5) and best edge feature (1&2′&4&5&6&7′&8).

Task 1	Recall	Precision	*F*-score
Strict evaluation mode	46.23	62.84	53.27
Approximate span and recursive mode	49.69	67.48	57.24
Event decomposition in the approximate span mode	51.19	73.21	60.25

Task 2	Recall	Precision	*F*-score

Strict evaluation mode	44.90	61.11	51.77
Approximate span and recursive mode	48.41	65.81	55.79
Event decomposition in the approximate span mode	50.52	73.15	59.76

Recall = TP/(TP + FN), Precision = TP/(TP + FP), and *F*-score = 2((Recall × Precision)/(Recall + Precision)).

**Table 6:** Comparison of *F*-score performance in Task 1 with other systems (under primary criterion: approximate span and recursive).

	Bjorne et al. 2009 [13]	Bjorne et al. 2011 [15]	Bjorne et al. 2012 [16]	TEES1.01	Riedel et al. 2011 [14]	Ours
*F*-score (%)	51.95	52.86	53.15	55.65	56.00	57.24

**Table 7:** The best feature combination after choosing features dynamically in trigger and edge detection.

	1	2	3	4	5	6
Feature in trigger	#Sentence feature	#Main feature	#Linear order	#Content feature	#Attached edge	#Chain feature
			Feature		Feature	
Feature size	18998	24944	73744	8573	100561	178345
(Merely one feature analysis)						
*F*-score under one class	0	42.05	3.50	27.11	7.33	5.48
Average contribution	0	0.001	4*e* − 5	0.003	7*e* − 5	3*e* − 5
(Double feature combination analysis)						
Best performance	(+4)	(+4)	(+4)	(+1)	(+2)	(+4)
Involving *i*th feature	45.39	36.65	22.29	45.39	28.27	23.48
Worst performance	(+2)	(+1)	(+1)	(+3)	(+1)	(+1)
Involving *i*th feature	0	0	0	22.29	0.91	3.10
(Three-feature combination analysis)						
Best performance	(+4, 5)	(+1, 4)	(+1, 4)	(+1, 5)	(+1, 4)	(+1, 4)
Involving *i*th feature	49.44	47.82	45.13	49.44	49.44	45.51
Worst performance	(+2, 3)	(+1, 3)	(+1, 2)	(+3, 6)	(+1, 3)	(+1, 3)
Involving *i*th feature	0.11	0.11	0.11	20.76	1.98	2.40
(Four-feature combination analysis)						
Best performance	(+2, 4, 5)	(+1, 4, 5)	(+1, 4, 5)	(+1, 2, 5)	(+1, 2, 4)	(+1, 2, 4)
Involving *i*th feature	50.71	50.71	49.74	50.71	50.71	48.82
Worst performance	(+2, 3, 6)	(+1, 3, 6)	(+1, 2, 6)	(+3, 5, 6)	(+1, 2, 3)	(+1, 2, 3)
Involving *i*th feature	5.77	5.77	5.77	21.13	7.02	5.77
(Five-feature combination analysis)						
Performance						
Without *i*th feature	34.22	47.1	49.90	16.37	50.19	51.34

**Table 8:** Analysis of average contribution of lexical features.

	Feature	Feature class	*F*-score	Feature size	Average contribution
1	Nonstem	Main feature	6.43	154	0.04175
2	POS	Main feature	1.52	47	0.03234
3	dt	Content feature	20.13	1172	0.01718
4	tt	Content feature	27.63	7395	0.00374
5	Stem	Main feature	35.45	11016	0.00322
