# Supplementary material for: A Novel Feature Selection Strategy for Enhanced Biomedical Event Extraction Using the Turku System
Source: Biomed Res Int. 2014 Apr 6;2014:205239. doi: 10.1155/2014/205239 (PMC3997098; doi:10.1155/2014/205239)
Supplement: Supplementary file 1 — There are five appendix files in the Supplementary Material. Appendix A is the feature generation rule for trigger and edge features. Appendix B is the result of feature combination experiment, which aim to analyze the trigger feature or edge feature contribution. Appendix C is the quantitative algorithm for evaluating contribution of feature class, while Appendix D is the feature combination experiment with modified features. Finally, Appendix E is feature combination experiment with modified features, which targeting on the ontribution analysis of sole trigger or edge feature. [file 205239.f1.pdf]

Appendix A

(Feature generation rule)

Table A1. Trigger features

Feature name	Def	Example
#Sentence feature		
bow	Token count	bow_-induced
nameCount	Count of token	nameCount_9
ne_bow	Count of token which satisfies isName = True	ne_bow_ATF-2
#main features		
POS	About part of speech	POS_MD
txt	Txt content of token	txt_A-activated:
nonstem	Remaining part after Porter stemmer	nonstem_ation
stem	Stem got by Porter Stemmer	nonstem_ancies
#linearorder feature		
linear	About linear order of the sentence which contains this token	linear_-1_POS_CC<br>linear_-1_txt_'nuclea'<br>linear_-1_isName<br>linear_-1_annType_protein
#content feature		
upper	Letter case	upper_case_middle<br>upper_case_start
has	Digital info of token	has_digits<br>has_fslash<br>has_hyphen<br>has_hyphenated_digit
dt	Double letter in token	dt_s+
tt	Triple letter in token	tt_lop
#attached edge feature		
t1Hin	In edge	t1HIn_Extinction
t1HOut	Out ege	t1HOut_Surfactant
#chain feature		
chain	Dependency information along with its direction info in chains	chain_dist_dist_1-frw_advcl-frw_conj_and-rev_prep_in
dep	Distance char in chain	dep_dist_dist_1conj_with
dist	Distance char in chain	dist_3_txt_densitometry

Table A2. Edge features

Feature name	Def	Example
#EntityFeatures		
e1_txt<br>e2_txt	Txt of Nodes info	e1_txt_transfection
e1_POS<br>e2_POS	Pos of nodes info	e1_POS_NNS
e1_annType<br>e2_annType	Class info of nodes	e1_annType_e1_Entity

e1_strength<br>e2_strength	Normalization value of annytype	e1_strength_Binding
e1_InteractionWord_e2_	isName=true of false for nodes	e1_InteractionWord_e2_Entity<br>e1_InteractionWord_e2_InteractionWord
eTypes	Anntype of nodes	eTypes_Binding_Binding
selfLoop	selfLoop=1 if the two nodes is the one	
#PathLengthFeatures		
len	Length of the shortest path	
len_tokens	Length value of the shortest path	len_tokens_15
#TerminusTokenFeature<br>(It could be omitted, since they are totally repeate in #EntityFeatures)		
#SingleElementFeature		
dep_Forward<br>dep_Reverse	Dependency between e1 and e2	dep_Reverse_appos
internalPOS<br>internalTxt<br>internalDep	Count thee POS, txt, dep info in the shortest path.	internalPOS_AUX<br>internalTxt_CAMs<br>internalDep_abbrev
#PathGram		
tokenPath	annotation type in the shortest path between e1 and e2.	tokenPath_Binding_e1_Binding___Binding__
tok	Pos, txt between e1 and e2, along with direction of dependency	tok_FFFFPOS_AUX<br>tok_FFFFtxt_Addition<br>tok_FFFFannType_Binding
depGram	Dep between e1 and e2, along with direction of dependency	depGram_RFFF_nsubj_prep_for_prep_of_amod
dep_[RF]{2,4}[0-3]	Dep info in a sliding windows between e1 and e2, along with direction of dependency	dep_RR0_prep_upon<br>dep_RR1_dep
edge_directions	The direction of dependency in the shorted path	edge_directions_FFFFFFFFF
other	Annotation type info in the shorted path	e1_Binding_depGram_FFFF_abbrev_nsubjpass_amod_hyphen_Protein
#PathEdgeFeature		
txt	The existence of txt	txt_weak
POS	The existence of POS	POS_VB
annType	annotation type in the shortest path	annType_e1_Binding
gov	Txt, POS and anntype in the adjacent nodes in the shortest path	gov_NNPS_JJ
triple	triple_Binding_advcl_Binding:	triple_Binding_advcl_Binding:
dep	Dep info in the adjacent nodes in the shortest path	Dep_prep_over
#SentenceFeature		
count	Count the occurrence of Anntype in the sentence which contain the shortest path.	count_Transcription
#GENIA		
GENIA	One is for entity1: isname=false<br>Second is e1 s isname=ture<br>Third is e2 s isname=false<br>Forth is e2 isnmae=ture	GENIA_regulation_of_event<br>GENIA_regulation_of_protein<br>GENIA_nested_event<br>GENIA_target_protein

Appendix B

(Feature combination experiment)

Table B1. Trigger feature contribution with combination

#One feature class			
Name of feature classes	F-score(%)	Size of features	Average contribution
1	None	18998	0
2	42.05	24944	0.001685776
3	3.5	73744	4.74615E-05
4	27.11	8573	0.003162254
5	7.33	100561	7.28911E-05
6	5.48	178345	3.0727E-05
#Two feature class combination			
Name of feature classes	F-score(%)	Size of features	Average contribution
1&2	0	43942	0
1&3	0	92742	0
1&4	45.39	27571	0.001646295
1&5	0.91	119559	7.6113E-06
1&6	3.1	197343	1.57087E-05
2&3	24.38	98688	0.000247041
2&4	36.65	33517	0.001093475
2&5	28.27	125505	0.00022525
2&6	16.76	203289	8.24442E-05
3&4	22.29	82317	0.000270782
3&5	5.94	174305	3.40782E-05
3&6	4.2	252028	1.66648E-05
4&5	27.57	109134	0.000252625
4&6	23.48	186918	0.000125617
5&6	7.56	278906	2.71059E-05
#Three feature class combination			
Name of feature classes	F-score(%)	Size of features	Average contribution
1&2&3	0.11	117686	9.34691E-07
1&2&4	47.82	52515	0.000910597
1&2&5	6.35	144503	4.39437E-05
1&2&6	5.85	222287	2.63173E-05
1&3&4	45.13	101315	0.000445442
1&3&5	1.98	193303	1.0243E-05
1&3&6	2.24	271087	8.26303E-06
1&4&5	49.44	128132	0.000385852
1&4&6	45.51	205916	0.000221012
1&5&6	7.5	297904	2.51759E-05
2&3&4	41.05	107261	0.000382711
2&3&5	21.27	199249	0.000106751
2&3&6	16.26	277033	5.86934E-05
2&4&5	35.42	134078	0.000264175
2&4&6	33.03	211862	0.000155903
2&5&6	17.05	303850	5.61132E-05
3&4&5	24.38	182878	0.000133313
3&4&6	20.76	260662	7.96434E-05
3&5&6	5.96	352650	1.69006E-05
4&5&6	23.35	287479	8.12233E-05
#Four feature class combination			
Name of feature classes	F-score(%)	Size of features	Average contribution
1&2&3&4	49.16	126259	0.000389358
1&2&3&5	7.02	218247	3.21654E-05
1&2&3&6	5.77	296031	1.94912E-05

1&2&4&5	50.71	153076	0.000331273
1&2&4&6	48.82	230860	0.00021147
1&2&5&6	15.47	322848	4.79173E-05
1&3&4&5	49.74	201876	0.000246389
1&3&4&6	46.7	279660	0.000166988
1&3&5&6	7.48	371648	2.01266E-05
1&4&5&6	46.73	306477	0.000152475
2&3&4&5	38.12	377594	0.000100955
2&3&4&6	36.33	285606	0.000127203
2&3&5&6	16.96	377594	4.4916E-05
2&4&5&6	32.47	312423	0.00010393
3&4&5&6	21.13	361223	5.84957E-05
#Five feature class combination			
Name of feature classes	F-score(%)	Size of features	Average contribution
1&2&3&4&5	51.34	226820	0.000226347
1&2&3&4&6	50.19	304604	0.000164771
1&2&3&5&6	16.37	396592	4.12767E-05
1&2&4&5&6	49.9	331421	0.000150564
1&3&4&5&6	47.1	380221	0.000123875
2&3&4&5&6	34.22	386167	8.86145E-05
#Six feature class combination			
Name of feature classes	F-score(%)	Size of features	Average contribution
1&2&3&4&5&6	51.21	405165	0.000126393

Table B2. Edge feature contribution with combination

#One feature class			
Name of feature classes	F-score(%)	Size of features	Average contribution
1	27.97	4313	0.00648505
2	0	25	0
4	17.67	2593	0.0068145
5	18.45	441684	4.1772E-05
6	13.09	25038	0.00052281
7	7.83	33	0.23727273
8	23.03	4	5.7575
#Two feature class combination			
Name of feature classes	F-score(%)	Size of features	Average contribution
1&2	18.55	4338	0.00427616
1&4	24.17	6906	0.00349986
1&5	24.19	445997	5.4238E-05
1&6	22.09	29351	0.00075261
1&7	32.91	4346	0.00757248
1&8	23.46	4317	0.00543433
2&4	0	2618	0
2&5	0	441709	0
2&6	22.42	25063	0.00089455
2&7	0	58	0
2&8	0	29	0
4&5	27.15	444277	6.1111E-05
4&6	15.48	27631	0.00056024
4&7	14.68	2626	0.00559025
4&8	26.43	2597	0.01017713
5&6	13	466722	2.7854E-05
5&7	18.2	441717	4.1203E-05
5&8	27.42	441688	6.208E-05
6&7	10.84	25071	0.00043237
6&8	12.66	25042	0.00050555
7&8	5.06	37	0.13675676
#Three feature class combination			
Name of feature classes	F-score(%)	Size of features	Average contribution
1&2&4	23.03	6931	0.00332275

1&2&5	31.98	446022	7.17E-05
1&2&6	44.2	29376	0.00150463
1&2&7	2.85	4371	0.00065202
1&2&8	25.69	4342	0.00591663
1&4&5	25.51	448590	5.6867E-05
1&4&6	19.4	31944	0.00060731
1&4&7	29.88	6939	0.0043061
1&4&8	24.43	6910	0.00353546
1&5&6	8.26	471035	1.7536E-05
1&5&7	22.66	446030	5.0804E-05
1&5&8	23.28	446001	5.2197E-05
1&6&7	24.5	29384	0.00083379
1&6&8	15.66	29355	0.00053347
1&7&8	18.74	4350	0.00430805
2&4&5	25.92	444302	5.8339E-05
2&4&6	30.53	27656	0.00110392
2&4&7	0	2651	0
2&4&8	0	2622	0
2&5&6	42.01	466747	9.0006E-05
2&5&7	3.4	441742	7.6968E-06
2&5&8	0	441713	0
2&6&7	18.51	25096	0.00073757
2&6&8	18	25067	0.00071808
2&7&8	0	62	0
4&5&6	8.84	469315	1.8836E-05
4&5&7	29.14	444310	6.5585E-05
4&5&8	30.45	444281	6.8538E-05
4&6&7	19.45	27664	0.00070308
4&6&8	17.25	27635	0.00062421
4&7&8	26.31	2630	0.0100038
5&6&7	14.26	466755	3.0551E-05
5&6&8	7.69	466726	1.6476E-05
5&7&8	28.11	441721	6.3637E-05
6&7&8	17.95	25075	0.00071585
#Four feature class combination			
Name of feature classes	F-score(%)	Size of features	Average contribution
1&2&4&5	38.84	448615	8.6578E-05
1&2&4&6	44.51	31969	0.00139229
1&2&4&7	13.84	6964	0.00198736
1&2&4&8	31.17	6935	0.00449459
1&2&5&6	46.1	471060	9.7864E-05
1&2&5&7	28.96	446055	6.4925E-05
1&2&5&8	37.21	446026	8.3426E-05
1&2&6&7	41.74	29409	0.00141929
1&2&6&8	44.08	29380	0.00150034
1&2&7&8	8.6	4375	0.00196571
1&4&5&6	20.05	473628	4.2333E-05
1&4&5&7	28.76	448623	6.4107E-05
1&4&5&8	24.42	448594	5.4437E-05
1&4&6&7	23.34	31977	0.0007299
1&4&6&8	19.01	31948	0.00059503
1&4&7&8	24.22	6943	0.00348841
1&5&6&7	20.78	471068	4.4113E-05
1&5&6&8	7.89	471039	1.675E-05
1&5&7&8	18.84	446034	4.2239E-05
1&6&7&8	1.8	29388	6.1249E-05
2&4&5&6	46.13	469340	9.8287E-05
2&4&5&7	8.18	444335	1.841E-05
2&4&5&8	23.92	444306	5.3837E-05
2&4&6&7	23.8	27689	0.00085955
2&4&6&8	39.53	27660	0.00142914
2&4&7&8	0	2655	0
2&5&6&7	35.26	466780	7.5539E-05
2&5&6&8	47.42	466751	0.0001016

2&5&7&8	0	441746	
2&6&7&8	10.19	25100	0.00040598
4&5&6&7	2.33	469348	4.9643E-06
4&5&6&8	17.78	469319	3.7885E-05
4&5&7&8	32.1	444314	7.2246E-05
4&6&7&8	6.24	27668	0.00022553
5&6&7&8	10.84	466759	2.3224E-05
#Five feature class combination			
Name of feature classes	F-score(%)	Size of features	Average contribution
1&2&4&5&6	49.02	473653	0.00010349
1&2&4&5&7	35.65	448648	7.9461E-05
1&2&4&5&8	39.6	448619	8.8271E-05
1&2&4&6&7	43.96	32002	0.00137366
1&2&4&6&8	50.23	31973	0.00157101
1&2&4&7&8	23.85	6968	0.00342279
1&2&5&6&7	44.19	471093	9.3803E-05
1&2&5&6&8	51.18	471064	0.00010865
1&2&5&7&8	34.37	446059	7.7053E-05
1&2&6&7&8	41.78	29413	0.00142046
1&4&5&6&7	22.81	473661	4.8157E-05
1&4&5&6&8	18.97	473632	4.0052E-05
1&4&5&7&8	25.81	448627	5.7531E-05
1&4&6&7&8	14.4	31981	0.00045027
1&5&6&7&8	5.4	471072	1.1463E-05
2&4&5&6&7	42.79	469373	9.1164E-05
2&4&5&6&8	49.26	469344	0.00010496
2&4&5&7&8	3.7	444339	8.327E-06
2&4&6&7&8	26.31	27693	0.00095006
2&5&6&7&8	44.17	466784	9.4626E-05
4&5&6&7&8	11.28	469352	2.4033E-05
#Six feature class combination			
Name of feature classes	F-score(%)	Size of features	Average contribution
1&2&4&5&6&7	47.29	473686	9.9834E-05
1&2&4&5&6&8	51.81	473657	0.00010938
1&2&4&5&7&8	36.85	448652	8.2135E-05
1&2&4&6&7&8	50.33	32006	0.00157252
1&2&5&6&7&8	51.29	471097	0.00010887
1&4&5&6&7&8	14.22	473665	3.0021E-05
2&4&5&6&7&8	48.32	469377	0.00010294
#Seven feature class combination			Average contribution
1&2&4&5&6&7&8	52.16	473690	0.000110114
#Eight feature class combination			Average contribution
1&2&3&4&5&6&7&8	51.21	477834	0.000107171

Appendix C
(Quantitative algorithm for evaluating contribution of feature class)

Table C1. Testing combination with Trigger features with fixed Edge features

\	Rank	Fixed Trigger features Testing combination with Edge features	AEE1(1)	AEE1(2)	AEE1(3)	AEE1(4)	AEE1(5)	AEE1(6)
51.34	1	1&2&3&4&5	1	1	1	1	1	0
51.21	2	1&2&3&4&5&6	1	1	1	1	1	0.5
50.71	3	1&2&4&5	1	1	0.667	1	1	0.3333
50.19	4	1&2&3&4&6	1	1	0.75	1	0.75	0.5
49.9	5	1&2&4&5&6	1	1	0.6	1	0.8	0.6
49.74	6	1&3&4&5	1	0.8333	0.667	1	0.833	0.5
49.44	7	1&4&5	1	0.7143	0.571	1	0.857	0.4286
49.16	8	1&2&3&4	1	0.75	0.625	1	0.75	0.375
48.82	9	1&2&4&6	1	0.7778	0.556	1	0.667	0.4444
47.82	10	1&2&4	1	0.8	0.5	1	0.6	0.4
47.1	11	1&3&4&5&6	1	0.7273	0.545	1	0.636	0.4545
46.73	12	1&4&5&6	1	0.6667	0.5	1	0.667	0.5
46.7	13	1&3&4&6	1	0.6154	0.538	1	0.615	0.5385
45.51	14	1&4&6	1	0.5714	0.5	1	0.571	0.5714
45.39	15	1&4	1	0.5333	0.467	1	0.533	0.5333
45.13	16	1&3&4	1	0.5	0.5	1	0.5	0.5
42.05	17	2	0.941	0.5294	0.471	0.941	0.471	0.4706
41.05	18	2&3&4	0.889	0.5556	0.5	0.944	0.444	0.4444
38.12	19	2&3&4&5	0.842	0.5789	0.526	0.947	0.474	0.4211
36.65	20	2&4	0.8	0.6	0.5	0.95	0.45	0.4
36.33	21	2&3&4&6	0.762	0.619	0.524	0.952	0.429	0.4286
35.42	22	2&4&5	0.727	0.6364	0.5	0.955	0.455	0.4091
34.22	23	2&3&4&5&6	0.696	0.6522	0.522	0.957	0.478	0.4348
33.03	24	2&4&6	0.667	0.6667	0.5	0.958	0.458	0.4583
32.47	25	2&4&5&6	0.64	0.68	0.48	0.96	0.48	0.48
28.27	26	2&5	0.615	0.6923	0.462	0.923	0.5	0.4615
27.57	27	4&5	0.593	0.6667	0.444	0.926	0.519	0.4444
27.11	28	4	0.571	0.6429	0.429	0.929	0.5	0.4286
24.38	29	2&3	0.552	0.6552	0.448	0.897	0.483	0.4138
24.38	30	3&4&5	0.533	0.6333	0.467	0.9	0.5	0.4
23.48	31	4&6	0.516	0.6129	0.452	0.903	0.484	0.4194
23.35	32	4&5&6	0.5	0.5938	0.438	0.906	0.5	0.4375
22.29	33	3&4	0.485	0.5758	0.455	0.909	0.485	0.4242
21.27	34	2&3&5	0.471	0.5882	0.471	0.882	0.5	0.4118
21.13	35	3&4&5&6	0.457	0.5714	0.486	0.886	0.514	0.4286
20.76	36	3&4&6	0.444	0.5556	0.5	0.889	0.5	0.4444
17.05	37	2&5&6	0.432	0.5676	0.486	0.865	0.514	0.4595
16.96	38	2&3&5&6	0.421	0.5789	0.5	0.842	0.526	0.4737
16.76	39	2&6	0.41	0.5897	0.487	0.821	0.513	0.4872
16.37	40	1&2&3&5&6	0.425	0.6	0.5	0.8	0.525	0.5
16.26	41	2&3&6	0.415	0.6098	0.512	0.78	0.512	0.5122
15.47	42	1&2&5&6	0.429	0.619	0.5	0.762	0.524	0.5238
7.56	43	5&6	0.419	0.6047	0.488	0.744	0.535	0.5349
7.5	44	1&5&6	0.432	0.5909	0.477	0.727	0.545	0.5455
7.48	45	1&3&5&6	0.444	0.5778	0.489	0.711	0.556	0.5556
7.33	46	5	0.435	0.5652	0.478	0.696	0.565	0.5435
7.02	47	1&2&3&5	0.447	0.5745	0.489	0.681	0.574	0.5319
6.35	48	1&2&5	0.458	0.5833	0.479	0.667	0.583	0.5208
5.96	49	3&5&6	0.449	0.5714	0.49	0.653	0.592	0.5306
5.94	50	3&5	0.44	0.56	0.5	0.64	0.6	0.52
5.85	51	1&2&6	0.451	0.5686	0.49	0.627	0.588	0.5294
5.77	52	1&2&3&6	0.462	0.5769	0.5	0.615	0.577	0.5385
5.48	53	6	0.453	0.566	0.491	0.604	0.566	0.5472

4.2	54	3&6	0.444	0.5556	0.5	0.593	0.556	0.5556
3.5	55	3	0.436	0.5455	0.509	0.582	0.545	0.5455
3.1	56	1&6	0.446	0.5357	0.5	0.571	0.536	0.5536
2.24	57	1&3&6	0.456	0.5263	0.509	0.561	0.526	0.5614
1.98	58	1&3&5	0.466	0.5172	0.517	0.552	0.534	0.5517
0.91	59	1&5	0.475	0.5085	0.508	0.542	0.542	0.5424
0.11	60	1&2&3	0.483	0.5167	0.517	0.533	0.533	0.5333
0	61	1	0.492	0.5082	0.508	0.525	0.525	0.5246
0	62	1&2	0.5	0.5161	0.5	0.516	0.516	0.5161
0	63	1&3	0.508	0.5079	0.508	0.508	0.508	0.5079
		AEE1 value computation	40.83	39.938	32.99	52.23	36.12	30.086

Table C2. Testing combination with Edge features with fixed Trigger features

F-score (%)	Rank	Fixed Trigger features Testing combination with Edge features	AEE1(1)	AEE1(2)	AEE1(4)	AEE1(5)	AEE1(6)	AEE1(7)	AEE1(8)
52.16	1	1&2&4&5&6&7&8	1	1	1	1	1	1	1
51.81	2	1&2&4&5&6&8	1	1	1	1	1	0.5	1
51.29	3	1&2&5&6&7&8	1	1	0.6667	1	1	0.66667	1
51.18	4	1&2&5&6&8	1	1	0.5	1	1	0.5	1
50.33	5	1&2&4&6&7&8	1	1	0.6	0.8	1	0.6	1
50. 23	6	1&2&4&6&8	1	1	0.6667	0.6667	1	0.5	1
49.26	7	2&4&5&6&8	0.85714	1	0.7143	0.7143	1	0.42857	1
49.02	8	1&2&4&5&6	0.875	1	0.75	0.75	1	0.375	0.875
48.32	9	2&4&5&6&7&8	0.77778	1	0.7778	0.7778	1	0.44444	0.8889
47.42	10	2&5&6&8	0.7	1	0.7	0.8	1	0.4	0.9
47.29	11	1&2&4&5&6&7	0.72727	1	0.7273	0.8182	1	0.45455	0.8182
46.13	12	2&4&5&6	0.66667	1	0.75	0.8333	1	0.41667	0.75
46.1	13	1&2&5&6	0.69231	1	0.6923	0.8462	1	0.38462	0.6923
44.51	14	1&2&4&6	0.71429	1	0.7143	0.7857	1	0.35714	0.6429
44.2	15	1&2&6	0.73333	1	0.6667	0.7333	1	0.33333	0.6
44.19	16	1&2&5&6&7	0.75	1	0.625	0.75	1	0.375	0.5625
44.17	17	2&5&6&7&8	0.70588	1	0.5882	0.7647	1	0.41176	0.5882
44.08	18	1&2&6&8	0.72222	1	0.5556	0.7222	1	0.38889	0.6111
43.96	19	1&2&4&6&7	0.73684	1	0.5789	0.6842	1	0.42105	0.5789
42.79	20	2&4&5&6&7	0.7	1	0.6	0.7	1	0.45	0.55
42.01	21	2&5&6	0.66667	1	0.5714	0.7143	1	0.42857	0.5238
41.78	22	1&2&6&7&8	0.68182	1	0.5455	0.6818	1	0.45455	0.5455
41.74	23	1&2&6&7	0.69565	1	0.5217	0.6522	1	0.47826	0.5217
39.6	24	1&2&4&5&8	0.70833	1	0.5417	0.6667	0.95833	0.45833	0.5417
39.53	25	2&4&6&8	0.68	1	0.56	0.64	0.96	0.44	0.56
38.84	26	1&2&4&5	0.69231	1	0.5769	0.6538	0.92308	0.42308	0.5385
37.21	27	1&2&5&8	0.7037	1	0.5556	0.6667	0.88889	0.40741	0.5556
36.85	28	1&2&4&5&7&8	0.71429	1	0.5714	0.6786	0.85714	0.42857	0.5714
35.65	29	1&2&4&5&7	0.72414	1	0.5862	0.6897	0.82759	0.44828	0.5517
35.26	30	2&5&6&7	0.7	1	0.5667	0.7	0.83333	0.46667	0.5333
34.37	31	1&2&5&7&8	0.70968	1	0.5484	0.7097	0.80645	0.48387	0.5484
32.91	32	1&7	0.71875	0.9688	0.5313	0.6875	0.78125	0.5	0.5313
32.1	33	4&5&7&8	0.69697	0.9394	0.5455	0.697	0.75758	0.51515	0.5455
31.98	34	1&2&5	0.70588	0.9412	0.5294	0.7059	0.73529	0.5	0.5294
31.17	35	1&2&4&8	0.71429	0.9429	0.5429	0.6857	0.71429	0.48571	0.5429
30.53	36	2&4&6	0.69444	0.9444	0.5556	0.6667	0.72222	0.47222	0.5278
30.45	37	4&5&8	0.67568	0.9189	0.5676	0.6757	0.7027	0.45946	0.5405
29.88	38	1&4&7	0.68421	0.8947	0.5789	0.6579	0.68421	0.47368	0.5263
29.14	39	4&5&7	0.66667	0.8718	0.5897	0.6667	0.66667	0.48718	0.5128
28.96	40	1&2&5&7	0.675	0.875	0.575	0.675	0.65	0.5	0.5
28.76	41	1&4&5&7	0.68293	0.8537	0.5854	0.6829	0.63415	0.5122	0.4878
28.11	42	5&7&8	0.66667	0.8333	0.5714	0.6905	0.61905	0.52381	0.5
27.97	43	1	0.67442	0.814	0.5581	0.6744	0.60465	0.51163	0.4884
27.42	44	5&8	0.65909	0.7955	0.5455	0.6818	0.59091	0.5	0.5
27.15	45	4&5	0.64444	0.7778	0.5556	0.6889	0.57778	0.48889	0.4889
26.43	46	4&8	0.63043	0.7609	0.5652	0.6739	0.56522	0.47826	0.5
26.31	47	4&7&8	0.61702	0.7447	0.5745	0.6596	0.55319	0.48936	0.5106
26.31	48	2&4&6&7&8	0.60417	0.75	0.5833	0.6458	0.5625	0.5	0.5208
25.92	49	2&4&5	0.59184	0.7551	0.5918	0.6531	0.55102	0.4898	0.5102
25.81	50	1&4&5&7&8	0.6	0.74	0.6	0.66	0.54	0.5	0.52

25.69	51	1&2&8	0.60784	0.7451	0.5882	0.6471	0.52941	0.4902	0.5294
25.51	52	1&4&5	0.61538	0.7308	0.5962	0.6538	0.51923	0.48077	0.5192
24.5	53	1&6&7	0.62264	0.717	0.5849	0.6415	0.5283	0.49057	0.5094
24.43	54	1&4&8	0.62963	0.7037	0.5926	0.6296	0.51852	0.48148	0.5185
24.42	55	1&4&5&8	0.63636	0.6909	0.6	0.6364	0.50909	0.47273	0.5273
24.22	56	1&4&7&8	0.64286	0.6786	0.6071	0.625	0.5	0.48214	0.5357
24.19	57	1&5	0.64912	0.6667	0.5965	0.6316	0.49123	0.47368	0.5263
24.17	58	1&4	0.65517	0.6552	0.6034	0.6207	0.48276	0.46552	0.5172
23.92	59	2&4&5&8	0.64407	0.661	0.6102	0.6271	0.47458	0.45763	0.5254
23.85	60	1&2&4&7&8	0.65	0.6667	0.6167	0.6167	0.46667	0.46667	0.5333
23.8	61	2&4&6&7	0.63934	0.6721	0.623	0.6066	0.47541	0.47541	0.5246
23.46	62	1&8	0.64516	0.6613	0.6129	0.5968	0.46774	0.46774	0.5323
23.34	63	1&4&6&7	0.65079	0.6508	0.619	0.5873	0.47619	0.47619	0.5238
23.28	64	1&5&8	0.65625	0.6406	0.6094	0.5938	0.46875	0.46875	0.5313
23.03	65	8	0.64615	0.6308	0.6	0.5846	0.46154	0.46154	0.5385
23.03	66	1&2&4	0.65152	0.6364	0.6061	0.5758	0.45455	0.45455	0.5303
22.81	67	1&4&5&6&7	0.65672	0.6269	0.6119	0.5821	0.46269	0.46269	0.5224
22.66	68	1&5&7	0.66176	0.6176	0.6029	0.5882	0.45588	0.47059	0.5147
22.42	69	2&6	0.65217	0.6232	0.5942	0.5797	0.46377	0.46377	0.5072
22.09	70	1&6	0.65714	0.6143	0.5857	0.5714	0.47143	0.45714	0.5
20.78	71	1&5&6&7	0.66197	0.6056	0.5775	0.5775	0.47887	0.46479	0.493
20.05	72	1&4&5&6	0.66667	0.5972	0.5833	0.5833	0.48611	0.45833	0.4861
19.45	73	4&6&7	0.65753	0.589	0.589	0.5753	0.49315	0.46575	0.4795
19.4	74	1&4&6	0.66216	0.5811	0.5946	0.5676	0.5	0.45946	0.473
19.01	75	1&4&6&8	0.66667	0.5733	0.6	0.56	0.50667	0.45333	0.48
18.97	76	1&4&5&6&8	0.67105	0.5658	0.6053	0.5658	0.51316	0.44737	0.4868
18.84	77	1&5&7&8	0.67532	0.5584	0.5974	0.5714	0.50649	0.45455	0.4935
18.74	78	1&7&8	0.67949	0.5513	0.5897	0.5641	0.5	0.46154	0.5
18.55	79	1&2	0.68354	0.557	0.5823	0.557	0.49367	0.4557	0.4937
18.51	80	2&6&7	0.675	0.5625	0.575	0.55	0.5	0.4625	0.4875
18.45	81	5	0.66667	0.5556	0.5679	0.5556	0.49383	0.45679	0.4815
18.2	82	5&7	0.65854	0.5488	0.561	0.561	0.4878	0.46341	0.4756
18	83	2&6&8	0.6506	0.5542	0.5542	0.5542	0.49398	0.45783	0.4819
17.95	84	6&7&8	0.64286	0.5476	0.5476	0.5476	0.5	0.46429	0.4881
17.78	85	4&5&6&8	0.63529	0.5412	0.5529	0.5529	0.50588	0.45882	0.4941
17.67	86	4	0.62791	0.5349	0.5581	0.5465	0.5	0.45349	0.4884
17.25	87	4&6&8	0.62069	0.5287	0.5632	0.5402	0.50575	0.44828	0.4943
15.66	88	1&6&8	0.625	0.5227	0.5568	0.5341	0.51136	0.44318	0.5
15.48	89	4&6	0.61798	0.5169	0.5618	0.5281	0.51685	0.4382	0.4944
14.68	90	4&7	0.61111	0.5111	0.5667	0.5222	0.51111	0.44444	0.4889
14.4	91	1&4&6&7&8	0.61538	0.5055	0.5714	0.5165	0.51648	0.45055	0.4945
14.26	92	5&6&7	0.6087	0.5	0.5652	0.5217	0.52174	0.45652	0.4891
14.22	93	1&4&5&6&7&8	0.6129	0.4946	0.5699	0.5269	0.52688	0.46237	0.4946
13.84	94	1&2&4&7	0.61702	0.5	0.5745	0.5213	0.52128	0.46809	0.4894
13.09	95	6	0.61053	0.4947	0.5684	0.5158	0.52632	0.46316	0.4842
13	96	5&6	0.60417	0.4896	0.5625	0.5208	0.53125	0.45833	0.4792
12.66	97	6&8	0.59794	0.4845	0.5567	0.5155	0.53608	0.45361	0.4845
11.28	98	4&5&6&7&8	0.59184	0.4796	0.5612	0.5204	0.54082	0.45918	0.4898
10.84	99	6&7	0.58586	0.4747	0.5556	0.5152	0.54545	0.46465	0.4848
10.84	100	5&6&7&8	0.58	0.47	0.55	0.52	0.55	0.47	0.49
10.19	101	2&6&7&8	0.57426	0.4752	0.5446	0.5149	0.55446	0.47525	0.495
8.84	102	4&5&6	0.56863	0.4706	0.549	0.5196	0.55882	0.47059	0.4902
8.6	103	1&2&7&8	0.57282	0.4757	0.5437	0.5146	0.5534	0.47573	0.4951
8.26	104	1&5&6	0.57692	0.4712	0.5385	0.5192	0.55769	0.47115	0.4904
8.18	105	2&4&5&7	0.57143	0.4762	0.5429	0.5238	0.55238	0.47619	0.4857
7.89	106	1&5&6&8	0.57547	0.4717	0.5377	0.5283	0.5566	0.4717	0.4906
7.83	107	7	0.57009	0.4673	0.5327	0.5234	0.5514	0.47664	0.486
7.69	108	5&6&8	0.56481	0.463	0.5278	0.5278	0.55556	0.47222	0.4907
6.24	109	4&6&7&8	0.55963	0.4587	0.5321	0.5229	0.55963	0.47706	0.4954
5.4	110	1&5&6&7&8	0.56364	0.4545	0.5273	0.5273	0.56364	0.48182	0.5
5.06	111	7&8	0.55856	0.4505	0.5225	0.5225	0.55856	0.48649	0.5045
3.7	112	2&4&5&7&8	0.55357	0.4554	0.5268	0.5268	0.55357	0.49107	0.5089
3.4	113	2&5&7	0.54867	0.4602	0.5221	0.531	0.54867	0.49558	0.5044
2.85	114	1&2&7	0.55263	0.4649	0.5175	0.5263	0.54386	0.5	0.5
2.33	115	4&5&6&7	0.54783	0.4609	0.5217	0.5304	0.54783	0.50435	0.4957
1.8	116	1&6&7&8	0.55172	0.4569	0.5172	0.5259	0.55172	0.50862	0.5
0	117	2	0.54701	0.4615	0.5128	0.5214	0.54701	0.50427	0.4957

0	118	2&4	0.54237	0.4661	0.5169	0.5169	0.54237	0.5	0.4915
0	119	2&5	0.53782	0.4706	0.5126	0.521	0.53782	0.4958	0.4874
0	120	2&7	0.53333	0.475	0.5083	0.5167	0.53333	0.5	0.4833
0	121	2&8	0.52893	0.4793	0.5041	0.5124	0.52893	0.49587	0.4876
0	122	2&4&8	0.52459	0.4836	0.5082	0.5082	0.52459	0.4918	0.4918
0	123	2&5&8	0.52033	0.4878	0.5041	0.5122	0.52033	0.4878	0.4959
0	124	2&7&8	0.51613	0.4919	0.5	0.5081	0.51613	0.49194	0.5
0	125	2&4&7	0.512	0.496	0.504	0.504	0.512	0.496	0.496
0	126	2&4&7&8	0.50794	0.5	0.5079	0.5	0.50794	0.5	0.5
0	127	2&5&7&8	0.50394	0.5039	0.5039	0.5039	0.50394	0.50394	0.5039
		AEE1 value computation	77.5994	83.84	68.451	74.877	77.7612	56.0854	66.347

Appendix D

(Feature combination experiment with modified features)

Table D1. The combination experiment after choosing modified features in Trigger detection

	Original	4'	6'	4'&6'
**Title:Trigger_Features_Combination**		**ft**	**some-chain**	**Some-chain-ft**
**Trigger-c**	200000	250000	200000	250000
**Edge-c**	28000	28000	28000	28000
**Beta**	0.65	0.7	0.65	0.65
# One Feature class				
Name	F-score(%)	F-score(%)	F-score(%)	F-score(%)
1	0	0	0	0
2	42.05	37.11	40.49	40.5
3	3.5	3.16	3.67	3.67
4	27.11	29.25	28.36	28.43
5	7.33	7.43	11.29	11.29
6	5.48	5.15	3.05	3.05
# Two feature class Combination				
Name of feature classes	F-score(%)	F-score(%)	F-score(%)	F-score(%)
1&2	0	0	0	0
1&3	0	0.11	0.22	0.22
1&4	45.39	46.09	44.97	44.95
1&5	0.91	1	6.16	6.16
1&6	3.1	3.52	0.22	0.22
2&3	24.38	18.11	15.4	15.35
2&4	36.65	39.57	37.8	37.85
2&5	28.27	21.8	20.36	20.35
2&6	16.76	13.28	10.37	10.36
3&4	22.29	23.26	22.04	21.99
3&5	5.94	5.34	6.99	6.97
3&6	4.2	4.24	2.8	2.79
4&5	27.57	29.2	25.35	25.35
4&6	23.48	23.71	28.8	28.8
5&6	7.56	7.33	8.05	8.06
# Three feature class Combination				
Name of feature classes	F-score(%)	F-score(%)	F-score(%)	F-score(%)
1&2&3	0.11	0.11	0.22	0.22
1&2&4	47.82	47.45	47.04	47.07
1&2&5	6.35	4.05	11.34	11.34
1&2&6	5.85	4.35	1.2	1.2
1&3&4	45.13	46.53	46.05	46.05
1&3&5	1.98	2.43	7.41	7.41
1&3&6	2.24	2.01	1.13	1.13
1&4&5	49.44	49.66	47.81	47.76
1&4&6	45.51	46.2	46.92	47
1&5&6	7.5	8.02	6	6
2&3&4	41.05	42.26	38.23	38.27
2&3&5	21.27	16.66	17.24	17.21
2&3&6	16.26	13.72	9.98	9.99
2&4&5	35.42	37.17	33.57	33.58
2&4&6	33.03	34.47	37.4	37.43
2&5&6	17.05	13.72	16.14	16.14
3&4&5	24.38	24.76	21.33	21.3
3&4&6	20.76	20.87	24.33	24.35
3&5&6	5.96	5.86	5.92	5.92
4&5&6	23.35	24.09	25.35	25.55
# Four feature class Combination				
Name of feature classes	F-score(%)	F-score(%)	F-score(%)	F-score(%)
1&2&3&4	49.16	48.79	48.69	48.6

1&2&3&5	7.02	5.44	15.01	15.02
1&2&3&6	5.77	4.55	1.81	1.81
1&2&4&5	50.71	50.92	50.33	50.44
1&2&4&6	48.82	48.94	48.33	48.38
1&2&5&6	15.47	11.91	10.21	10.21
1&3&4&5	49.74	50.36	46.44	46.47
1&3&4&6	46.7	46.93	46.51	46.51
1&3&5&6	7.48	8.72	6.17	6.17
1&4&5&6	46.73	47.95	48.13	48.1
2&3&4&5	38.12	39.52	32.94	32.92
2&3&4&6	36.33	37.87	37.09	37.06
2&3&5&6	16.96	13.39	14.09	14.1
2&4&5&6	32.47	33.53	34	34.01
3&4&5&6	21.13	21.77	22.28	22.33
# Five feature class Combination				
Name of feature classes	F-score(%)	F-score(%)	F-score(%)	F-score(%)
1&2&3&4&5	**51.34**	**52.21**	51.61	51.61
1&2&3&4&6	50.19	50.57	50.14	50.14
1&2&3&5&6	16.37	14.19	11.45	11.45
1&2&4&5&6	49.9	51.03	51.15	51.05
1&3&4&5&6	47.1	48.37	48.12	48.13
2&3&4&5&6	34.22	36.18	33.64	33.64
# Six feature class Combination				
Name of feature classes	F-score(%)	F-score(%)	F-score(%)	F-score(%)
1&2&3&4&5&6	**51.21**	51.62	**51.93**	**51.99**

Table D2. The combination experiment after choosing modified features in Edge detection (with fixed best Trigger features)

		2'+best Trigger	7'+best Trigger	2'&7'+best Trigger
**Title:Edge_Features_Combination**	**Original**	**pathlength**	**sentence**	**pathlength-sentence**
**Trigger-c**	**200000**	250000	200000	250000
**Edge-c**	**28000**	20000	2000	2500
**beta**	**0.65**	0.7	0.7	0.7
# One feature class				
Name of feature classes	F-score(%)	F-score(%)	F-score(%)	F-score(%)
1	27.97	25.8	24.37	22.6
2	0	0	0	0
4	17.67	12.88	18.15	15.53
5	18.45	9.12	9.46	11.96
6	13.09	6.68	14.68	7.27
7	7.83	14.25	8.7	12.03
8	23.03	21.41	21.41	21.41
# Two feature class Combination				
Name of feature classes	F-score(%)	F-score(%)	F-score(%)	F-score(%)
1&2	18.55	24.47	28.15	27.05
1&4	24.17	22.2	21.65	13.25
1&5	24.19	6.08	9.15	4.12
1&6	22.09	5.15	15.51	5.3
1&7	32.91	25.96	24.42	25.15
1&8	23.46	21.88	22.51	21.96
2&4	0	0	0	0
2&5	0	0.33	4.83	1.18
2&6	22.42	22.57	23.79	23.05
2&7	0	0	0	0
2&8	0	0	0	0
4&5	27.15	31.35	29.15	31.46
4&6	15.48	5.31	5.71	5.71
4&7	14.68	17	15.12	12.78
4&8	26.43	27.4	28.11	27.43
5&6	13	3.89	4.25	3.16
5&7	18.2	22.45	21.94	12.5

5&8	27.42	33.15	32.68	32.33
6&7	10.84	5.94	10.46	7.05
6&8	12.66	15.61	17.79	6.28
7&8	5.06	15.4	9.58	12.16
# Three feature class Combination				
Name of feature classes	F-score(%)	F-score(%)	F-score(%)	F-score(%)
1&2&4	23.03	31.36	34.65	34.66
1&2&5	31.98	37.74	38.9	38.73
1&2&6	44.2	45.75	45.6	45.37
1&2&7	2.85	15.5	15.58	16.71
1&2&8	25.69	32.77	36.67	35.26
1&4&5	25.51	11.3	9.99	5.31
1&4&6	19.4	6.84	7.15	7.36
1&4&7	29.88	25.62	25.42	25.86
1&4&8	24.43	22.96	23.18	21.86
1&5&6	8.26	3.85	4.25	3.91
1&5&7	22.66	18.49	5.59	5.19
1&5&8	23.28	18.67	18.85	9.72
1&6&7	24.5	16.6	14.58	15.7
1&6&8	15.66	16.67	17.39	9.04
1&7&8	18.74	24.17	14.94	19.99
2&4&5	25.92	15.59	26.12	25.82
2&4&6	30.53	29.01	29.3	30.31
2&4&7	0	0	0	0
2&4&8	0	0	0	0
2&5&6	42.01	42.77	43.17	42.16
2&5&7	3.4	5.35	4.43	4.66
2&5&8	0	0.45	0	1.76
2&6&7	18.51	20.08	18.17	19.39
2&6&8	18	19.06	19.68	19.15
2&7&8	0	0	0	0
4&5&6	8.84	5.72	8.83	9.16
4&5&7	29.14	29.26	30.15	28.54
4&5&8	30.45	36.46	35	34.09
4&6&7	19.45	8.67	15.33	9.12
4&6&8	17.25	5.37	5.48	6.31
4&7&8	26.31	29.12	23.63	25.84
5&6&7	14.26	6.13	5.15	4.27
5&6&8	7.69	9.3	9.87	13.98
5&7&8	28.11	30.27	31.15	29.73
6&7&8	17.95	4.63	4.35	3.36
# Four feature class Combination				
Name of feature classes	F-score(%)	F-score(%)	F-score(%)	F-score(%)
1&2&4&5	38.84	42.7	43.35	43.38
1&2&4&6	44.51	45.18	44.67	44.61
1&2&4&7	13.84	25.83	25.64	27.23
1&2&4&8	31.17	37.66	38.36	38.71
1&2&5&6	46.1	49.16	48.47	48.45
1&2&5&7	28.96	35.87	35.38	35.22
1&2&5&8	37.21	38.03	39.23	39.25
1&2&6&7	41.74	44.41	44.24	43.89
1&2&6&8	44.08	49.82	50.03	49.56
1&2&7&8	8.6	25.98	25.31	25.4
1&4&5&6	20.05	5.96	6.06	5.13
1&4&5&7	28.76	21.42	11.91	8.91
1&4&5&8	24.42	27.99	27.97	23.06
1&4&6&7	23.34	12.61	17.06	10.01
1&4&6&8	19.01	9.07	17.41	8.56
1&4&7&8	24.22	24.29	10.5	19.99
1&5&6&7	20.78	6.61	5.47	5.7
1&5&6&8	7.89	5.27	8.99	4.9
1&5&7&8	18.84	18.27	7.59	16.14
1&6&7&8	1.8	15.81	8.34	6.12
2&4&5&6	46.13	47.23	47.88	47.14
2&4&5&7	8.18	8.27	8.32	9.6

2&4&5&8	23.92	24.19	26.31	25.38
2&4&6&7	23.8	24.64	23.57	23.98
2&4&6&8	39.53	34.2	40.05	39.71
2&4&7&8	0	0	0	0
2&5&6&7	35.26	37.03	34.64	34.45
2&5&6&8	47.42	48.21	47.94	47.61
2&5&7&8	0	0	0	0.45
2&6&7&8	10.19	17.57	13.64	15.07
4&5&6&7	2.33	5.83	6.35	5.92
4&5&6&8	17.78	14.75	23.43	20.69
4&5&7&8	32.1	33.8	36.41	33.83
4&6&7&8	6.24	5.75	5.85	9.27
5&6&7&8	10.84	7.55	8.46	3.38
# Five feature class Combination				
Name of feature classes	F-score(%)	F-score(%)	F-score(%)	F-score(%)
1&2&4&5&6	49.02	50.91	50.15	50.54
1&2&4&5&7	35.65	39.64	40.55	40.94
1&2&4&5&8	39.6	41.5	42.25	41.93
1&2&4&6&7	43.96	43.72	43.61	43.54
1&2&4&6&8	50.23	51.04	50.59	50.86
1&2&4&7&8	23.85	33.98	33.94	34.73
1&2&5&6&7	44.19	46.45	46.38	46.28
1&2&5&6&8	51.18	51.13	50.65	51.09
1&2&5&7&8	34.37	37.14	36.84	37.22
1&2&6&7&8	41.78	50.34	49.04	48.28
1&4&5&6&7	22.81	5.75	5.44	5.81
1&4&5&6&8	18.97	14.17	13.55	12.87
1&4&5&7&8	25.81	23.61	12.93	15.72
1&4&6&7&8	14.4	15.21	7.15	8.26
1&5&6&7&8	5.4	5.75	4.47	4.16
2&4&5&6&7	42.79	44.18	42.27	43.25
2&4&5&6&8	49.26	50.42	50.48	50.2
2&4&5&7&8	3.7	4.34	4.25	6.12
2&4&6&7&8	26.31	28.52	26.57	28.08
2&5&6&7&8	44.17	45.5	43.32	43.39
4&5&6&7&8	11.28	16.14	6.74	9.12
# Six feature class Combination				
Name of feature classes	F-score(%)	F-score(%)	F-score(%)	F-score(%)
1&2&4&5&6&7	47.29	49.64	49.36	49.13
1&2&4&5&6&8	51.81	52.29	51.44	51.85
1&2&4&5&7&8	36.85	38.86	39.04	39.18
1&2&4&6&7&8	50.33	51.79	51.7	51.81
1&2&5&6&7&8	51.29	51.61	51.45	51.65
1&4&5&6&7&8	14.22	15.09	4.13	5.11
2&4&5&6&7&8	48.32	48.82	48.17	48.1
# Seven class Combination				
Name of feature classes	F-score(%)	F-score(%)	F-score(%)	F-score(%)
1&2&4&5&6&7&8	**52.16**	**52.37**	**52.47**	**52.68**

Appendix E

(Feature combination experiment with modified features)

Table E2. Contribution of sole Trigger feature

#Sentence feature	f-score (%)	Feature size	Contribution of sole feature	
bow	N/A	15962		
nameCount	N/A	15		
ne_token	N/A	3021		
#main features				
POS	1.52	47	0.03234	★★
txt	35.5	13727	0.00259	★
nonstem	6.43	154	0.04175	★★
stem	35.45	11016	0.00322	★
#linear order				
linear	3.5	73744	4.7E-05	
#content				
upper	0	2	0	
has	0	4	0	
dt	20.13	1172	0.01718	★★
tt	27.63	7395	0.00374	★
#attached edge				
t1HIn	2.4	47410	5.1E-05	
t1HOut	7.69	53151	0.00014	
chain				
chain	5.47	136239	4E-05	
dep	3.57	1148	0.00311	★
dist	8.26	40958	0.0002	
total	51.21	405165	0.00013	

Table E2. Contribution of sole Edge feature

	Feature size	f-score (%)	Feature size	Contribution of sole feature	
#EntityFeatures	4319		4319		
e1_txt	1218				
e2_txt	2828	11.08	4046	0.002738507	
e1_POS	22				
e2_POS	25	8.32	47	0.177021277	
e1_annType	25				
e2_annType	26	46.44	51	0.910588235	★
e1_strength	31				
e2_strength	31	0	62	0	
e1_InteractionWord_e2_	2	27.31	2	13.655	★★
eTypes	110	13.91	110	0.126454545	
selfLoop	1	0	1	0	
#PathLengthFeatures	25				
len	1	0	1	0	
len_tokens	24	32.16	24	1.34	★
#TerminusTokenFeatures(thisfeature class is totally repeated with ones in #EntityFeatures)	4144		4144	0	
tokTerm1_txt	1218				
tokTerm2_txt	2828	11.08	4046	0.002738507	
tokTerm1_POS	22				
tokTerm2_POS	25	8.32	47	0.177021277	
tokTerm1_annType	25				
tokTerm2_annType	26	1.31	51	0.025686275	
#SingleElementFeatures	2593		2593	0	

dep_Forward	105				
dep_Reverse	123	6.85	228	0.03004386	
internalPOS	29	0	29	0	
internalTxt	2225	13.69	2225	0.006152809	
internalDep	111	1.31	111	0.011801802	
#PathGrams	441684		441684	0	
tokenPath	8009	N/A	8009	#VALUE!	
tok_	22543	6.81	22543	0.000302089	
depGram_	26897	18.41	26897	0.000684463	
dep_[RF]{2,4}[0-3]	3886	15.19	3886	0.003908904	
edge_directions	746	30.17	746	0.040442359	
Special	376797 + 2806	21.46	379603	5.65327E-05	
#PathEdgeFeature	25038		25038	0	
txt	2535	13.55	2535	0.005345168	
POS	32	18.18	32	0.568125	★
annType	33	1.67	33	0.050606061	
gov	15176	5.13	15176	0.000338034	
triple	7135	15.4	7135	0.002158374	
dep_	127	18.78	127	0.147874016	
#SentenceFeatures	33		33		
count	33	7.83	33	0.237272727	
#GENIA	4	23.03	4	5.7575	★★
total	477840				
